# The Many Ways by Which *O*-GlcNAcylation May Orchestrate the Diversity of Complex Glycosylations

**DOI:** 10.3390/molecules23112858

**Published:** 2018-11-02

**Authors:** James Biwi, Christophe Biot, Yann Guerardel, Anne-Sophie Vercoutter-Edouart, Tony Lefebvre

**Affiliations:** Unité de Glycobiologie Structurale et Fonctionnelle, Université de Lille, CNRS, UMR 8576, UGSF, 59000 Lille, France; james.biwi@univ-lille.fr (J.B.); christophe.biot@univ-lille.fr (C.B.); yann.guerardel@univ-lille.fr (Y.G.); anne-sophie.vercoutter@univ-lille.fr (A.-S.V.-E.)

**Keywords:** *O*-GlcNAc, *O*-GlcNAcylation, OGT, *O*-GlcNAc transferase, OGA, *O*-GlcNAcase, glycosylation

## Abstract

Unlike complex glycosylations, *O*-GlcNAcylation consists of the addition of a single *N*-acetylglucosamine unit to serine and threonine residues of target proteins, and is confined within the nucleocytoplasmic and mitochondrial compartments. Nevertheless, a number of clues tend to show that *O*-GlcNAcylation is a pivotal regulatory element of its complex counterparts. In this perspective, we gather the evidence reported to date regarding this connection. We propose different levels of regulation that encompass the competition for the nucleotide sugar UDP-GlcNAc, and that control the wide class of glycosylation enzymes via their expression, catalytic activity, and trafficking. We sought to better envision that nutrient fluxes control the elaboration of glycans, not only at the level of their structure composition, but also through sweet regulating actors.

## 1. Glycosylations Form a Huge Family of Co- and Post-Translational Modifications

**Figure 1:**
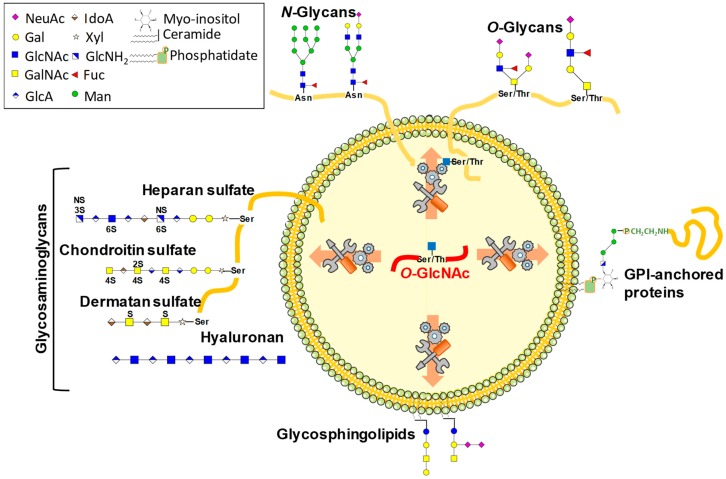
*O*-GlcNAcylation is a potential regulator of complex glycosylations.

**Figure 2:**
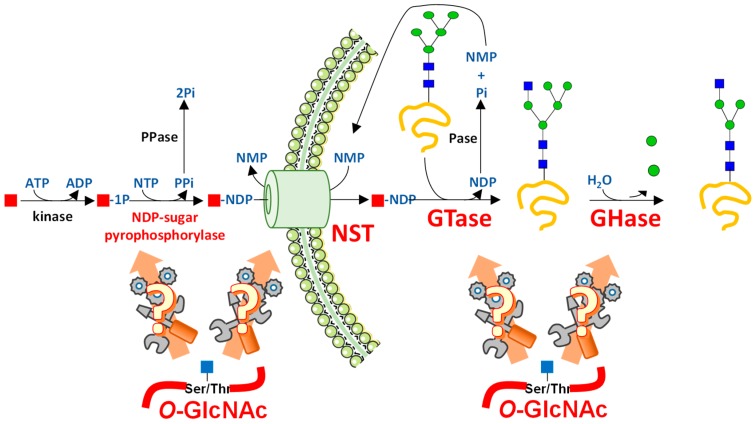
The different targets by which *O*-GlcNAcylation may govern complex glycosylation. Nucleotide-sugars are high-energy donors used in many glycosylation processes (see text for details). A carbohydrate (red square) is first phosphorylated by a kinase using ATP. The second step consists of the synthesis of NDP-sugar (mainly UDP-Glc, UDP-Gal, UDP-GlcNAc, UDP-GalNAc, UDP-GlA, UDP-Xyl, GDP-Man, and GDP-Fuc; or CMP-Sialic acid, not shown here) by an NDP-sugar pyrophosphorylase. Nucleotide sugars are used in the cytoplasm (as UDP-GlcNAc for OGT or HAS, as an example), or transported into organelles such as Golgi apparatus by nucleotide sugar transporters (NSTs). Nucleotide sugars are used by Glycosyltransferases (GTases) to build complex glycans. Glycosylhydrolases (GHases) release sugars from glycans, glycoproteins, or glycolipids (not shown). *O*-GlcNAcylation is likely to intervene with any of these steps.

Since the discovery that, during and following synthesis, proteins can undergo chemical modifications or proteolysis, several hundreds of co- and post-translational modifications (PTMs) have been described in all domains of life. These modifications endow living beings with an extra level of information to the genome, epigenome, transcriptome, and proteome. Among PTMs, glycosylations form a vast and heterogeneous family [1,2,3]. In humans, it is considered that around eighty percent of proteins are glycosylated in the diverse cell compartments, and that nearly two to four percent of the genome encode proteins involved in glycosylation processes [4,5]. Glycosylation is therefore an ensemble of spatially and temporally well-organized PTMs, which are matter- and energy-consuming. In contrast to complex glycosylations, including *N*-Glycosylproteins, mucins, and proteoglycans (Figure 1), *O*-GlcNAcylation consists of the transfer of a single *N*-acetylglucosamine moiety, through a β-linkage, onto serine and threonine residues of proteins confined within the nuclear, cytosolic, and mitochondrial compartments of cells [6,7,8]. Due to this subcellular distribution, which strikingly contrasts with the localization of proteins bearing complex glycans, it has been largely overlooked that *O*-GlcNAc and the other types of glycosylations are capable of interacting and interfering with each other (Figure 1). Knowing that complex glycosylations involve a whole set of steps, enzymes, and transporters, *O*-GlcNAcylation is likely to influence some of these processes (Figure 2).

## 2. *O*-GlcNAcylation Differs from Other Glycosylations in Many Ways

**Figure 3:**
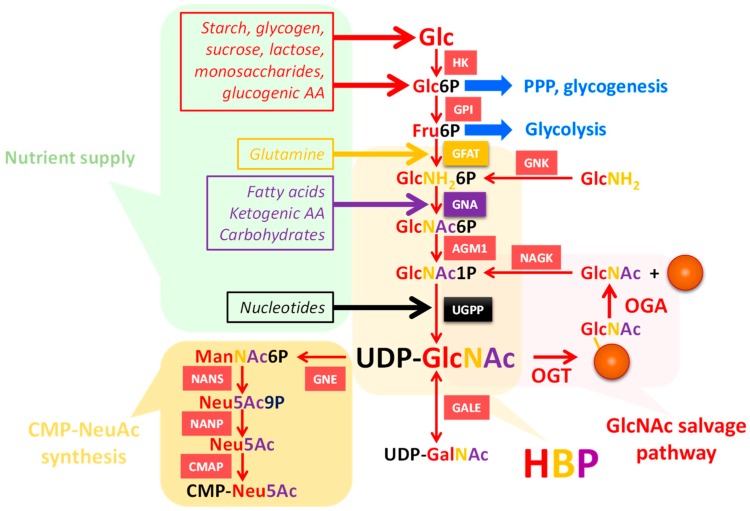
The high-energy donor UDP-GlcNAc is provided by the hexosamine biosynthetic pathway (HBP) and is connected to UDP-GalNAc and CMP-NeuAc. A fraction of the glucose enters the HBP for the production of UDP-GlcNAc, which is used for glycosylation processes, including *O*-GlcNAcylation. HBP is supplied by many sources of nutrients, making HBP flux an indicator of nutritional status. Glucosamine by-passes the rate-limiting enzyme of HBP, GFAT, and N-acetylglucosamine is recycled by the GlcNAc salvage pathway. Starting from UDP-GlcNAc, UDP-GalNAc is generated by epimerization, and CMP-NeuAc is synthesized through a series of enzymatic reactions. HK, hexokinase; GPI, glucose-6-phosphate isomerase; GFAT, glutamine:fructose-6-phosphate amidotransferase; GNA, GlcNH_2_-6-phosphate-N-acetyltransferase; AGM1, phospho-GlcNAc mutase; UGPP, UDP-GlcNAc pyrophosphorylase; NAGK, GlcNAc kinase; GNK, GlcNH_2_ kinase; OGT, *O*-GlcNAc transferase; OGA, *O*-GlcNAcase; GNE, UDP-GlcNAc 2-epimerase (+ManNAc kinase); NANS, NeuAc-9-phosphate synthase; NANP, NeuAc-9-phosphate phosphatase; CMAP, CMP-NeuAc synthase.

During the last decades, *O*-GlcNAcylation has emerged as a switch for protein function. Indeed, like phosphorylation, with which it can compete for the same or neighboring sites [8,9], *O*-GlcNAcylation is a dynamic and nutrient-dependent process [8,10] (Figure 3). *O*-GlcNAc cycling is controlled by two enzymes, the *O*-linked β-*N*-acetyl-d-glucosaminyltransferase (OGT or *O*-GlcNAc transferase: GT41 in CAZy classification [11]) and the *O*-linked β-*N*-acetyl-d-glucosaminidase (OGA or *O*-GlcNAcase: GH84 family in CAZy classification). OGT catalyzes the transfer of a GlcNAc residue from the activated substrate uridine-diphosphate-*N*-acetylglucosamine (UDP-GlcNAc), which is the end-product of the hexosamine biosynthetic pathway (HBP) [8,12]. In turn OGA hydrolyzes the GlcNAc moiety (Figure 3). In mammals, there are three isoforms of OGT, the nucleocytoplasmic (ncOGT), short (sOGT), and mitochondrial OGT (mOGT), and two isoforms of OGA have been described, the nucleocytoplasmic (ncOGA) and short (sOGA) OGAs [8]. A new form of OGT resident of the endoplasmic reticulum (ER), called EOGT, meaning EGF (epidermal growth factor) domain-specific OGT, was first characterized in *Drosophila* [13] and later in the pig *Sus scrofa* [14]. EOGT does not show any obvious homology with other forms of OGT, but it shares a 19.4% identity with the β-2-xylosyltransferase of *Arabidopsis thaliana*, suggesting that both enzymes share common structural features. Furthermore, *O*-GlcNAcylation performed by EOGT on EGF-like repeats of Notch1 can be further elongated into more complex glycans, which is also a noticeable difference with the nucleocytoplasmic *O*-GlcNAc [15].

*O*-GlcNAc modifies a huge variety of different proteins (more than one thousand have been identified to date), such that this PTM interferes at the molecular level with transcription, protein synthesis, proteasomal degradation, cell trafficking, and the organization of the cytoskeleton. Thanks to these molecular actions, *O*-GlcNAcylation regulates most of the fundamental cellular processes such as cell signaling, cell cycle, and apoptosis [8,16]. In turn, deregulation of *O*-GlcNAc cycling has been linked with the etiology of diabetes, cancers, cardiovascular diseases, and neuronal disorders [17]. Although apparently very different and distant from each other, it has been documented that disturbances of complex glycosylations are also observed for the same disorders [18,19]. Nevertheless, too few elements linking the control of complex glycosylations by *O*-GlcNAc have been reported. Here we propose a reflection on the control of complex glycosylations at the various levels that *O*-GlcNAcylation can exert.

## 3. UDP-GlcNAc Participates in Many Forms of Glycosylation

### 3.1. UDP-GlcNAc Is Produced by the HBP and Can Be Converted into UDP-GalNAc and CMP-NeuAc

UDP-GlcNAc is the end-product of the HBP (Figure 3), and is the second most abundant nucleotide-based structure in the cell after ATP. HBP not only depends on glucose and carbohydrate abundance, but also on lipids, amino-acids, nucleotides, and ATP [8,12,17]. HBP flux reflects nutrients and energy availability; UDP-GlcNAc is therefore considered a nutritional sensor, whose modulation affects not only *O*-GlcNAcylation, but also all other kinds of glycosylation, playing a central role in the regulation of cell properties, such as proliferation and differentiation. UDP-GlcNAc pools can be influenced by the *O*-GlcNAcylation state, as shown in the *Caenorhabditis. elegans* model where *ogt* and *oga* knock outs had perturbed nucleotide sugar levels [20].

From a nutritional stand-point, UDP-GlcNAc concentrations can be increased via enhanced glucose and glucosamine (which by-passes the rate-limiting HBP enzyme, GFAT) uptake. For example, in hyperglycemic cells there is an increase in the UDP-GlcNAc pool via HBP flux and, in turn, *O*-GlcNAcylation is upregulated [21]. When UDP-GlcNAc production was impeded, in min6 beta cells, via GFAT inhibition, *O*-GlcNAcylation was reduced, emphasizing the dependence of *O*-GlcNAcylation on the UDP-GlcNAc pool [22]. In addition, depletion of glucose in hematopoietic cells leads to the reduction of cellular UDP-GlcNAc concentrations [23]. There have also been reports showing that fatty acid supplementation could increase the UDP-GlcNAc concentration [24].

Regarding UDP-GlcNAc concentrations during cellular stress, there is some evidence that the nucleotide sugar concentration changes in HeLa and HepG2 when cells undergo nutritional stress, but is also perturbed under other stress inducers [25].

UDP-GlcNAc is used by OGT for the *O*-GlcNAcylation of nucleocytoplasmic and mitochondrial proteins (Figure 3) [5,8]. It is also used for the biosynthesis of various glycoconjugates: *N*- and *O*-glycoproteins, glycosaminoglycans (GAG), and glycosphingolipids (GSL) (Table 1). Transmembrane nucleotide sugar transporters are necessary to import UDP-GlcNAc into the lumen of the ER and Golgi apparatus, in which the other GlcNAc transferases are active. Of note, the Km of OGT for UDP-GlcNAc is estimated to be 545 nM [26]. This low Km confers an advantage to OGT rather than the organelle-resident GlcNAc transferases which display a much higher Km value for UDP-GlcNAc (from 0.04 mM for MGAT1 to 11 mM for MGAT5), thus requiring elevated UDP-GlcNAc levels inside these organelles.

The HBP also supplies a relatively large amount of UDP-GalNAc, which is generated by the epimerization of UDP-GlcNAc by the cytosolic enzyme GALE (UDP-GalNAc-4-epimerase) [27] (Figure 3). UDP-GalNAc is mainly used in the ER and Golgi apparatus for glycan biosynthesis. A fraction of UDP-GlcNAc also generates the nucleotide sugar CMP-NeuAc that is used in the Golgi apparatus by the sialyltransferases. This requires a more complex metabolic pathway involving the rate-limiting enzyme UDP-GlcNAc 2-epimerase/ManNAc kinase (GNE/MNK) [28] (Figure 3). Thus, glycan structures containing GalNAc and sialic acids are highly susceptible to regulate, or be regulated by, *O*-GlcNAcylation by competing with upstream metabolites.

### 3.2. UDP-GlcNAc Is a Single Substrate of Many Suitors

#### 3.2.1. *N*-Glycosylation

UDP-GlcNAc is used for the initiation step of *N*-glycosylation in the ER, and for *N*-glycan branching in the Golgi apparatus [29]. This nucleotide sugar is the substrate for the first two steps of *N*-glycosylation processing (Table 1) that take place in the ER and end with the synthesis of the lipid-linked oligosaccharide Glc_3_Man_9_GlcNAc_2_-PP-dolichol, which is transferred en bloc onto nascent polypeptide chains by oligosaccharyltransferase (OST) complex. DPAGT1 or Alg7 (Asn-linked glycosylation) catalyzes the formation of the GlcNAc-PP-dolichol from UDP-GlcNAc and dolichol-phosphate (Dol-P). Then GlcNAc_2_-PP-dolichol is generated by the utilization of a second molecule of UDP-GlcNAc, catalyzed by the bipartite enzyme Alg13/14 UDP-GlcNAc transferase. UDP-GlcNAc is also utilized in the *N*-glycan synthesis steps occurring in the Golgi apparatus, namely by the GlcNAc transferases MGAT1, MGAT2, MGAT3, MGAT4, and MGAT5. The latter is responsible for β-1,6-branched structures (poly-LacNAc), and is more active in cancer and involved in metastasis (Table 1). In this respect, it has been shown that dietary GlcNAc, delivered orally, is capable of stimulating the uptake of nutrients by enhancing the UDP-GlcNAc pool and the branching of β-1,6-*N*-glycans in the Golgi apparatus [30]. The poly-LacNAc chains are built by the sequential activity of the GlcNAc transferase B3GNT8 [5,31] (Table 1) and the β4-GalT (β-1,4-galactosyltransferase).

#### 3.2.2. *O*-Glycosylation

*O*-glycans, that include mucins (mucin-type *O*-glycans), represent another large family of glycosylations, in which a GalNAc residue is usually attached to a serine or a threonine of the peptide backbone in the Golgi apparatus [1,32]. Among the large variety of monosaccharides found on *O*-Glycans (Gal, GalNAc, Fuc, and NeuAc), the GlcNAc contribution is non-negligible. In particular, the GlcNAc residue is part of cores 2, 3, and 4; C2GnT is responsible for the β-1,6 linkage of the GlcNAc onto the GalNAc, and C3GnT transfers GlcNAc through a β-1,3 linkage to generate the Core 3 that is further processed into Core 4 through the activity of C4GnT (Table 1). It is noteworthy that the expression of C3GnT is low in colon tumors and seems absent in cancer cultured-cells [33].

#### 3.2.3. Lewis Antigens

UDP-GlcNAc is indirectly involved in the synthesis of the terminal Lewis blood group system found at the terminal side chains of *N*- and *O*-glycans (Table 1). These determinants are generated from the fucosylation of type-1 (Galβ-1,3-GlcNAc) and type-2 (Galβ-1,4-GlcNAc) structures [1,5]. Depending on whether a second fucose is incorporated or not, Lewis^a/x^ or Lewis^b/y^ are built. Then, sialylation of Lewis^a/x^ can occur to give sialyl-Lewis^a/x^.

#### 3.2.4. Glycosaminoglycans

Hyaluronan (HA) is a non-sulfated glycosaminoglycan made of disaccharide repeat units of GlcNAc and Glucuronic acid [(GlcNAc β-1,4 GlcA)β-1,3] (Figure 1). Contrary to the biosynthesis of the sulfated glycosaminoglycans, such as heparan sulfate, chondroitin sulfate, and keratin sulfate, that occur in the Golgi apparatus, the hyaluronan synthases (HAS1–3) are embedded in the plasma membrane. HAS are synthesized in the Golgi apparatus and then targeted to the plasma membrane to be activated. Therefore, HA is extruded out of the cell as the glycosyltransferases work. Due to its high-molecular weight and its abundance in animals, hyaluronan biosynthesis is a high consumer of UDP-GlcNAc, making this process a rheostat of the use of UDP-GlcNAc which may have indirect repercussions on *O*-GlcNAcylation levels, as discussed in Hascall et al. [34]. Also, and of particular interest, the stability, transcription, and trafficking of hyaluronan synthases are under the control of *O*-GlcNAcylation [35,36,37]. Vigetti and collaborators showed that *O*-GlcNAcylation of HAS2 at Ser221 greatly increases its stability in primary human aortic smooth muscle cells [35]. Similarly, HAS3 is *O*-GlcNAcylated in melanoma cells and enhanced *O*-GlcNAcylation reduced its lysosomal degradation [36]. Furthermore, it was shown that *O*-GlcNAcylation levels positively regulate the mRNA transcription of both HAS2 and its natural antisense regulatory transcript HAS2-AS1 [37]. This occurs via the recruitment of the p65 NFκB transcription factor to promoters, thus facilitating the opening of the chromatin of the HAS2 promoter in a HAS2-AS1-dependent manner [37]. Finally, the trafficking of HAS3 is affected by both UDP-GlcNAc and UDP-GlcA availability [36]. UDP-GlcNAc maintains HAS3 at the cell membrane, and this characteristic can be recapitulated by increased *O*-GlcNAcylation. Together these findings point out a close relationship between HA synthesis and the dynamics of *O*-GlcNAcylation. HAS and OGT use the same UDP-GlcNAc cytoplasmic pool, thus these enzymes are in direct competition for the substrate. However, HAS and OGT display a distinct Km for UDP-GlcNAc; 400 µM [38] and 545 nM [26], respectively. Thanks to this unusual high affinity for UDP-GlcNAc, OGT is able to be active even in the presence of low nucleotide sugar concentrations, or in the case of a high consumption of UDP-GlcNAc arising from the synthesis of HA.

The biosynthesis of the other classes of GAG—heparin, heparan sulfate (EXT family of GTases [39]), chondroitin sulfate, dermatan sulfate, and keratin sulfate—are also high consumers of UDP-GlcNAc, or of its epimer UDP-GalNAc (Figure 1) (Table 1). Nevertheless, no relationship between sulfated GAG and *O*-GlcNAcylation has been documented clearly yet. The only observation is that the elevation of UDP-GlcNAc and *O*-GlcNAcylation induced by glucosamine increased the synthesis of chondroitin sulfate in human aortic smooth muscle cells [35].

#### 3.2.5. Glycosphingolipids and Glypiation

Glycosphingolipids (GSLs) are a subclass of glycolipids particularly enriched in the membranes of all living beings (Figure 1). Some of these GSLs have GlcNAc residues in their composition, particularly the neutral core lacto- and neolacto-series, and predominantly in the invertebrate series Mollu and Arthro [1]. UDP-GlcNAc is also crucial for the initiation of the biosynthesis of glycosylphosphatidylinositol (GPI) at the cytosolic face of the ER membrane; a GlcNAc residue is transferred from UDP-GlcNAc to phosphatidylinositol (α-1,6-GlcNAc) by the GPI-GlcNAc transferase complex, made of seven different subunits (PIG-A/C/H/P/Q/Y and DPM2) [40] (Table 1). Then the GlcNAc residue is further deacetylated into glucosamine. To date, a potential crosstalk between GSL biosynthesis and *O*-GlcNAcylation still remains to be fully explored.

## 4. Interfering with *O*-GlcNAc Cycling Disrupts Production of Nucleotide Sugars through Expression of HBP Enzymes

Hanover’s lab reported that the production of the nucleotide sugars UDP-Glc and UDP-HexNAc was perturbed in the worm model *C. elegans* lacking either OGT or OGA [20]. *Ogt-1* and *oga-1* null animals exhibited higher GalNAc, GlcNAc, and Gal when compared to wild-type animals for PNGase A and F resistant glycans, while there was no significant difference found for PNGase F sensitive glycans. This suggests that in this model, *N*-glycan biosynthesis is less dependent on *O*-GlcNAc homeostasis than other glycoconjugates. Interestingly, in *ogt-ko* animals, the mRNA levels of the genes encoding for enzymes of the HBP *gfat-2* (glutamine:fructose-6-phosphate amidotransferase-2), *gna-2* (glucosamine-6-phosphate N-acetyltransferase-2) and C36A4.4 (a putative UDP-GlcNAc pyrophosphorylase) (Figure 3) were more elevated, explaining in part the increase in UDP-HexNAc. This suggests that interfering with *O*-GlcNAc homeostasis has repercussions on the global glycosylation by tuning the synthesis and the availability of the nucleotide sugar donors. These observations were later corroborated by Zhong and collaborators who identified the HBP enzymes UAP1 (UDP-*N*-acetylhexosamine pyrophosphorylase-1) and GNPDA1 (Glucosamine-6-phosphate isomerase-1) to be upregulated in OGT null MEFs (mouse embryonic fibroblasts) when compared with wild-type MEFs [41]. However, these authors did not report any UDP-HexNAc assays, which would have strengthened the idea that OGT knock-down could increase the synthesis of nucleotide sugar in mammalian cells.

## 5. *O*-GlcNAcylation Widely and Finely Orchestrates Gene Expression

It has been long known that OGT tightly interferes with gene expression, as exemplified by its partners and substrates involved in transcriptional regulation, epigenetics, and chromatin topology (e.g., Myc, HIC1, β-catenin, HCF1, C-terminal domain of RNA polymerase II, TET, mSin3A, HDAC, HIRA, MLL5, RING1B, EZH2, LSD2, etc.; for exhaustive review see [42]). In this respect, it is remarkable that the Polycomb group (PcG) protein Supersexcomb (Sxc) was identified as the homolog of OGT in *Drosophila* [43]. Sxc *O*-GlcNAcylates polyhomeotic, which is a member of the Polycomb repressive complex 1 (PRC1) involved in transcriptional repression.

Gene expression is controlled by epigenetic modifications of DNA (e.g., methylation of CpG islands) and histones by combination between PTMs, such as methylation, acetylation, phosphorylation, ubiquitination, SUMOylation, and, as more recently proposed, *O*-GlcNAcylation [44], to generate the complex histone code [45]. Later, further evidence demonstrated the role of OGT and *O*-GlcNAcylation in the regulation of gene expression through the TET (Ten-eleven translocation) proteins [46,47,48,49]. The TET protein family is comprised of 5-methylcytosine hydroxylase proteins that reverse the gene silencing activity of DNA-methyltransferases (DNMT). In 2013, three groups independently reported a dialog between TET proteins and OGT, in which the former promotes the recruitment of the latter to CpG-rich TSS (transcription start site) that corresponds to transcriptionally active genes [47,48,49]. Vella and collaborators determined regions of association with OGT, throughout the mouse embryonic stem cells (mESCs) genome, by chromatin immunoprecipitation coupled to high-throughput DNA sequencing (ChIP-seq) with specific anti-OGT antibodies [48]. They showed that OGT associates throughout the mESC genome on 11,552 binding sites, among which 62% of the OGT binding sites are found within promoters. Interestingly, in mESCs transiently depleted from OGT, the expression of several genes involved in *N*- and *O*-glycan biosynthesis were either upregulated or downregulated (Table 2). Since then, few researchers have pushed forward this field of investigation to detail how *O*-GlcNAcylation regulates gene expression of GTases, glycosylhydrolases (GHases), or any other protein implicated in glycosylation.

Additionally, a recent study has highlighted a link between OGT and the glycosidase MAN1A1 (Mannosidase alpha class 1A) in cancer cells [50]. Cancer progression is often associated with an increase in surface *N*-glycans [51,52,53], and, on the other hand, OGT and *O*-GlcNAcylation are known to be up-regulated in carcinogenesis [5]. Studying cholangiocarcinoma, Phoomak and collaborators previously showed that high glucose stimulates the expression of hexokinase-2, the HBP rate-limiting enzyme GFAT, and OGT, contributing to the metastatic properties of these cells through elevated levels of *O*-GlcNAcylation [54]. Recently, they unveiled an interesting mechanistic pathway by which *O*-GlcNAcylation actively promotes metastasis [50]. The authors first mapped the *N*-glycome on membranes of cholangiocarcinoma cells, in which OGT was silenced, and revealed an increased level of biantennary complex and decreased high-mannose *N*-linked glycans. Further analyses indicated that silencing OGT perturbed the activation of the PI3K and MAPK signaling pathways, and in turn allowed the accumulation of the transcription factor FOXO3, a downstream target of the mitogenic pathways. MAN1A1, the enzyme responsible for the trimming of high-mannose structures in the ER, is a target gene of FOXO3, elucidating the mechanism by which OGT indirectly controls *N*-glycosylation found at the surface of cancer cells.

## 6. *O*-GlcNAcylation Regulates Protein Expression

**Figure 4:**
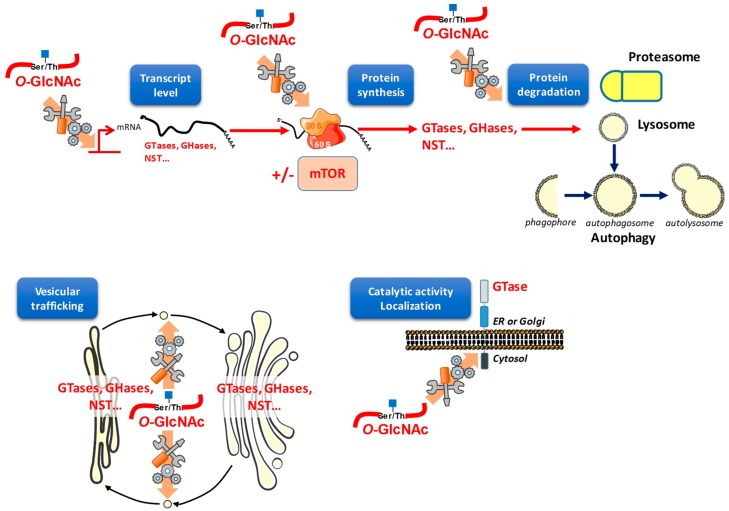
*O*-GlcNAcylation is a regulator of the most fundamental biological processes, such as protein fate, traffic, localization, and activity. Many studies report the functions played by *O*-GlcNAcylation in transcription, translation (especially in a mTOR-dependent manner), and protein degradation. The different patterns of glycosylation are likely to be regulated by these fundamental processes. Via vesicular trafficking, *O*-GlcNAcylation could also coordinate GTases, GHases, NSTs, or any other actors involved in glycosylation distribution, by targeting these actors to specific organelles. Lastly, since many glycosylation enzymes that are resident in the ER and the Golgi apparatus are integral proteins, their cytoplasmic tail might be a platform of interaction with OGT.

When the protein steady-state level is perturbed, cell homeostasis is deregulated. If a protein is in excess it can be toxic and when it aggregates, amyloidosis or neuropathologies can emerge. *O*-GlcNAcylation is capable of interfering with protein content, both at the synthesis and at the degradation levels (Figure 4).

### 6.1. O-GlcNAc Assists Protein Translation

The first evidence of *O*-GlcNAcylation interference with protein biosynthesis was from Datta and collaborators [55,56]. eIF-2 (eukaryotic peptide chain initiation factor 2) associates with the protein p67. Interestingly, eIF-2 is protected from inactivation specifically by the *O*-GlcNAc forms of p67, reducing its phosphorylation [55,56]. Later, it was shown that nearly half of ribosomal proteins are *O*-GlcNAcylated, and that both OGT and OGA strongly associate with ribosomes [57]. Overexpression of OGT or OGA in HepG2 cells affects the homeostasis of ribosomal subunits, indicating that *O*-GlcNAc cycling may be important for the maturation and assembly of ribosomes [57]. Since *N*-glycosylation starts in the ER, with the en bloc transfer of a precursor glycan to the nascent protein by the oligosaccharyltransferase (OST) [1,58], we hypothesize that perturbation in *O*-GlcNAc levels therefore indirectly impact the rate of *N*-glycosylation. It should also be noted that, similar to *N*-glycosylation, *O*-GlcNAc glycosylation can occur in a co-translational manner [59]. In this case, *O*-GlcNAcylation helps in the stabilization of nascent proteins to prevent their premature ubiquitin-dependent proteasomal degradation [59].

*O*-GlcNAcylation modifies many ribosomal proteins, including RPS6, that are part of the mTOR pathway [57]. While the exact function of *O*-GlcNAcylation on the translational machinery, including ribosomal proteins, is yet to be clarified, more studies have investigated the link between mTOR signaling, which is directly implicated in the control of translation, and *O*-GlcNAcylation [60,61,62]. OGT and mTOR pathways are both dependent on nutrient availability, and mutual regulation of both pathways has been highlighted [60]. The first evidence of *O*-GlcNAcylation regulation by mTOR was independently reported by Cho [61] and Reginato [62] groups. Inhibition of mTOR blocks OGT expression and, conversely, elevated OGA protein levels, as a consequence of a decrease in *O*-GlcNAcylation [61]. Recently, our team revealed that pharmacological inhibition of OGA reverberates on mTOR activation in the colon cancer cell line HCT116 [62]. The roles of mTOR signaling in the regulation of protein translation, metabolism of nucleotides and lipids, and the inhibition of autophagy are well known [63,64,65], but the involvement of mTOR in complex glycosylation processes remains to be deciphered. To date, only two studies, from the same group, have reported a connection between the Akt/mTOR signaling and the expression of sialyltransferases; mTOR activation is positively correlated with ST3GAL6 expression in hepatoma carcinoma cells [66], but is negatively correlated with ST8SIA4 expression in follicular thyroid cancer cells [67]. To our knowledge, there is no other data available on the regulation of the expression of glycosylation enzymes by the translation factors or the mTOR pathway.

### 6.2. O-GlcNAc May Interfere with Protein Degradation through Different Pathways

Protein degradation occurs through three main routes: the lysosomal and proteasomal pathways, and the nutrient-dependent process autophagy (Figure 4). The lysosome is an acidic intracellular compartment budding from the late Golgi apparatus, in which nonspecific proteases degrade proteins of the secretory pathway which must be expelled from the ER and the Golgi apparatus or that are mislocalized. The lysosome is also involved in the degradation of cell surface proteins that undergo endocytosis. Lysosomal machinery can also degrade cytosolic proteins by invagination of the lysosomal membrane (exosome-like vesicles). The proteasome mainly degrades cytosolic proteins in a ubiquitin-dependent manner, but also degrades misfolded proteins from the secretory pathway that have been retrotranslocated in the cytoplasm by a process called ERAD (ER-associated degradation). Lastly, autophagy is a catabolic process by which cells deliver cytoplasmic material for degradation into lysosomes. Autophagy is also induced by nutrient starvation in order to provide metabolite recycling for stressed cells. For the autophagic process, a double membrane engulfs cytosolic components, and the resulting autophagosomes fuse with lysosomes (Figure 4).

In the ER, misfolded glycoproteins are transported to the cytosol, polyubiquitinated, and destined for proteasomal degradation [68,69]. The catabolism of glycoproteins is also ensured by lysosomes. In these organelles, glycoproteins are digested into amino acids and monosaccharides by different lysosomal proteases and glycosidases [70]. More recently, autophagy has been shown to be involved in the efficient catabolism of cytoplasmic free *N*-glycans [71]. In turn, glycoconjugates regulate autophagosome maturation, as recently reviewed [72]. Since the different actors of the glycosylation processes are distributed throughout the cell, each degradation pathway is likely to contribute to their homeostasis and turnover.

The regulation of the proteasomal degradation of nucleocytoplasmic proteins by *O*-GlcNAcylation is widely studied [73,74]. On the one hand, the 26S proteasome is directly inhibited by *O*-GlcNAc modification of the Rpt2 ATPase in the 19S cap [75]. On the other hand, *O*-GlcNAcylation is also in a complex interplay with ubiquitin that tags proteins for degradation [76]. Many proteins are directly protected against proteasomal degradation by *O*-GlcNAcylation, as exemplified by Sp1, p53, Myc, estrogen receptors, β-catenin, FAS, or Clock. Intriguingly, OGT stability is regulated by the E3 ubiquitin ligase LSD2 in cancer cells [77]. Although much less documented than the proteasomal pathway, a few studies report the impact of *O*-GlcNAcylation on autophagy [78]. First, OGT protein level is affected when autophagic degradation is induced following mTOR inhibition in HepG2 cells, suggesting that OGT stability is also regulated by autophagy [61]. In addition, in a fly model of Huntington disease, downregulation of *O*-GlcNAcylation increases autophagy by promoting lysosome–autophagosome fusion in the neuronal cells and eye imaginal discs of *Drosophila*, thus partially restoring eye morphology and vision [78]. The negative regulation of autophagy flux by *O*-GlcNAcylation has been demonstrated also in *C. elegans* and mammalian cells, in which OGT depletion promotes autophagosome maturation [79]. Some cellular mechanisms have been deciphered. SNAP-29 is a protein of the SNARE complex, which mediates the fusion of autophagosomes with lysosomes in mammalian cells. The *O*-GlcNAcylation of SNAP-29 impairs the interaction of SNAP-29 with its SNARE complex partners, syntaxin 17 (stx17) and lysosomal VAMP8 (vesicle-associated membrane protein 8), thus blocking the autophagic flux [79]. GRASP55 (or GORASP2) is a Golgi apparatus stacking protein, crucial for autophagy, whose *O*-GlcNAcylation is glucose-dependent [80]. The decreased *O*-GlcNAcylation of GRASP55 upon glucose deprivation promotes its localization at the interface between autophagosome and lysosome membranes, thus favoring the autophagic flux by the fusion of organelles in HeLa cells [80,81]. However, it has been shown that nutrient starvation in mouse liver induces an increase in OGT activity, leading to the *O*-GlcNAcylation and activation of two key proteins involved in the autophagy initiation, Ulk1 and Ulk2 [82]. Taken together these studies indicate that, depending on the factors that induce autophagy and cell types, *O*-GlcNAcylation can exert either positive or negative effect on autophagic flux to integrate the nutrient availability with autophagy. Since autophagy implicates vesicle formation and fusion, it is likely that OGT, OGA, and *O*-GlcNAcylation interfere with vesicular trafficking of the proteins involved in glycosylation processes. 

## 7. *O*-GlcNAcylation Orchestrates Vesicular Trafficking and Therefore May Redistribute Glycosylation Enzymes

The complex glycosylation pathways require intricate trafficking of vesicles between organelles of the endomembrane system (Figure 4) [4,32]. Perturbation of this traffic results in incomplete or defective glycosylation, inducing protein misfolding and toxicity, and may ultimately result in cell death. To support such a correct pattern of glycosylation, GTases, GHases, and NSTs must be targeted to the correct compartment. For that purpose, eukaryotic cells have developed a sophisticated system of vesicle transport in which coat proteins assemble. The cargo is selected and transported into a budded vesicle toward its target organelle (ER, Golgi apparatus, endosomes, lysosome, or plasma membrane) or secreted in the extracellular space.

PTMs, such as phosphorylation and ubiquitination, play a critical role in vesicular trafficking, particularly with anterograde traffic involving the multiprotein coatomer COP (coat protein) II. COPII is specialized in the transport between the ER and the Golgi apparatus, this process being naturally blocked during mitosis [83]. More recently there has been evidence that *O*-GlcNAc PTM also plays a part in the regulation of the anterograde traffic. SEC24P, a component of COPII, is *O*-GlcNAcylated during interphase but not during mitosis [84]. SEC23A, SEC24C, SEC31A, and TFG (Trk-fused gene that assists SEC functions) were also recognized as bearing *O*-GlcNAc residues [85]. It was therefore proposed that this modification may alter and stop COPII trafficking when cells enter division. Interestingly, *O*-GlcNAcylation regulates COPII cargo trafficking by mediating COPII component interactions [86]. This finding is supported by a defect of collagen trafficking in a model of zebrafish, in which specific sites of *O*-GlcNAcylation of Sec23A were mutated [87]. COPI enables transport within the Golgi apparatus and from the cis-Golgi network back to the ER (retrograde transport). But to our knowledge, the potential regulation of COPI-mediated trafficking by *O*-GlcNAcylation has not been studied yet.

Vesicular trafficking also involves architectural proteins forming the cytoskeleton, on which vesicles and organelles dock to move [86]. We recently showed that down-regulating OGT modifies cell shape features of the fetal human colon cell line CCD841CoN [87]. OGT silencing did not modify the expression of actin, but we proposed that actin-binding proteins may be up- or down-regulated when OGT was silenced. Indeed, in breast cancer cells, OGT promotes invasion in a cofilin-dependent manner, and *O*-GlcNAcylation of cofilin at Ser108 localizes this actin-interacting protein to the invadopodia [88]. So, under conditions of varying OGT expression or activity, actin may itself be affected, either indirectly or directly (since actin is, itself, *O*-GlcNAcylated [89,90]), by altered *O*-GlcNAcylation. *O*-GlcNAcylation was also widely studied on tubulin, another major component of the cytoskeleton network. *O*-GlcNAcylation of α-tubulin reduces heterodimerization of α- and β-tubulins, and *O*-GlcNAcylated forms of tubulins are unable to polymerize into microtubules [91]. These observations suggest that the default of *O*-GlcNAcylation of both actin and tubulin networks might impede vesicular trafficking, and therefore perturb the distribution of proteins involved in glycosylation. Experimental evidences are needed to confirm this hypothesis.

Furthermore, *O*-GlcNAcylation blocks the transport of the adherens junction-based protein E-cadherin to the plasma membrane [92]. E-cadherin is first synthesized as a precursor of 140 kDa in the ER. During its traffic along the secretory pathway, E-cadherin must be processed by Furin, which cleaves a part of the N-terminal region, to be properly folded. E-cadherin is also glycosylated; *N*-glycans are found on the extracellular domains 4 and 5 at Asn554/566/618/633, and *O*-GlcNAcylation modifies the cytoplasmic tail [93]. Several *N*-glycans regulate E-cadherin adherent properties [93,94]. For example, a glycosylation defect at Asn633 decreases the stability of E-cadherin, while a default of glycosylation at Asn554 and Asn566 causes a defect in calcium-dependent adherence [95]. Regarding *O*-GlcNAcylation of E-cadherin, it seems that it impinges on the exit of E-cadherin from the ER, suggesting that this PTM might influence the *N*-glycosylation pattern of E-cadherin by controlling its trafficking between the ER and the Golgi apparatus [92].

Since the *N*-glycosylation of plasma membrane proteins controls folding, processing, stability, and interaction with partners, *O*-GlcNAcylation should also indirectly affect behaviors of *N*-glycosylproteins, such as transporters or growth factor receptors [23,96]. This statement is strengthened by the observation that glucose deprivation reduces growth factor signaling by decreasing the flux of HBP [23]. Also, many GTases resident of the ER and the Golgi apparatus exhibit cytoplasmic tails, which might offer interaction surfaces for OGT and OGA, consequently undergoing *O*-GlcNAcylation and de-*O*-GlcNAcylation cycling (Figure 4). This hypothesis deserves investigations and could open a new mode of regulation of complex glycosylations. It might also provide alternative explanations as to why and how glycosylations are often affected in pathologies, including cancers and neurodegenerative diseases in which *O*-GlcNAc dynamics are disturbed.

## 8. Future Directions

In recent decades, technological advances in terms of structural analysis and molecular biology have raised awareness of the complexity and wide variety of glycosylation processes throughout living beings. *O*-GlcNAcylation, however, stands out from its counterparts. This glycosylation could not be much simpler from a structural point of view as only one monosaccharide is in question. Additionally, *O*-GlcNAcylation subcellular distribution (i.e., nucleocytoplasmic and mitochondrial) seems to be distant and distinct from *O*- and *N*-glycosylations or GAGs, which are all confined within intracellular organelles or secreted (outside the cell). However, *O*-GlcNAcylation and complex glycosylations are likely to be subject to variations in the same pathological disorders suggesting that common elements bring them together (Table 3). Studies have revealed that the crosstalk between these two glycosylation worlds is in fact not so slight. Although, the transcriptional and translational regulation of enzymes, and other actors involved in complex glycosylation, is well understood, we are only beginning to understand those of *O*-GlcNAcylation enzymes, OGT, and OGA [97,98]. Nevertheless, in view of the involvement of *O*-GlcNAcylation in the processes of gene expression [42,46,47,48,49,50,55,56,57,58,59,60,61,62], there is strong evidence to believe that part of complex glycosylation patterns can be controlled by *O*-GlcNAcylation at these levels. This research path seems a priority with the advent of CHIP-Seq and transcriptome analysis that, with deep sequencing, have revolutionized life sciences. This type of approach should allow the understanding of the impact of *O*-GlcNAcylation deregulation on transcript levels of complex glycosylation enzymes. At the level of translational control, things seem to be even more unclear, since the translational control of GTases, GHases, NSTs, and other actors of glycosylation processes seem even more obscure than their control at the mRNA level. 

Except for the biosynthesis of HA, the processes of complex glycosylation proceed in the intracellular organelles (ER, Golgi apparatus, etc.), which require a very precise and finely regulated vesicular trafficking [4]. Indeed, traffic disorders result in the poor addressing of glycosylation enzymes and nucleotide sugar transporters, resulting in abnormal glycosylation patterns. This vesicle trafficking is directed by the cytoskeleton and coordinated by a variety of specialized factors, such as COP, SNARE, SNAP, and small G-proteins (Rab in particular). More studies have pointed to the disorganization of microtubules and microfilaments under perturbed *O*-GlcNAcylation conditions. This may partly explain the impact on membrane and vesicular network dependent glycosylations; also, it appears that COPII plays a major role in this redistribution of vesicular content [83,84,85,86]. On the other hand, regarding the retrograde transport in which COPI is involved, no study has focused yet on the potential role of *O*-GlcNAcylation. Small G-proteins could also actively interfere with vesicular trafficking processes. Although several proteomic studies identified the *O*-GlcNAcylation of small G-proteins, the functional relevance of this PTM has to be deciphered.

There are nine main nucleotide sugars required for the various forms of glycosylation [4]. These nucleotide sugars are synthesized by a set of enzymes located in the cytoplasm, and are transported in organelles where the corresponding GTases reside. Thus, a better understanding of the role of *O*-GlcNAcylation on the activity of the enzymes activating sugars and transporters should shed some light on the disturbances of glycosylation patterns in response to a failure of *O*-GlcNAcylation. Unfortunately, currently there is no simple, sensitive, robust, and inexpensive technique for the detection and quantification of nucleotide sugars. Nevertheless, there is reason to believe that, in the coming years, thanks to biotechnological advances in the various fields of biochemistry, cellular and molecular biology and biophysics, a whole body of studies focusing on the regulation and deregulation of complex glycosylation processes by *O*-GlcNAcylation, will flourish. This should make it possible to better understand the variations of glycosylation observed in various pathologies, and perhaps to better control and edit them. In that sense, it was recently shown that OGT, itself, is mutated in XLID (X-linked intellectual disability) [99,100]. It is of particular interest to know whether patients suffering this pathology display aberrant complex glycosylations profiles, reinforcing the relationship between the two kinds of PTM.

Lastly, glycogen, although built up using UDP-Glc and not UDP-GlcNAc, could be a good model for studying the putative cross-regulation between polysaccharide storage and *O*-GlcNAcylation. Particularly this could be achieved through the glycosylation of the metabolic pathway enzymes, for example glycogen synthase [101,102]. Of particular interest is the observation done by Cho’s laboratory [103]. The authors revealed that, under glucose deprivation, glycogenolysis occurs to provide the cell UDP-GlcNAc. This supply of the OGT substrate is permitted due to the activation of glycogen phosphorylase and GFAT. In turn, the level of the rate-limiting enzyme of the glycolysis, PFK1, is decreased. This study is a nice example of a molecular dialog between two different metabolic pathways. Therefore, it will be of interest, in the near future, to examine the interplay between nutrients, nucleotide sugars, energy storage, and *O*-GlcNAcylation.

## Figures and Tables

**Table 1:** Main enzymes competing for UDP-GlcNAc.

Symbol	Designation	Glycosylation Process	Subcellular Localization	EC Number	CAZy
OGT	*O*-linked β-*N*-acetylglucosaminyltransferase	*O*-GlcNAcylation	Cyt., nucl., mit.	2.4.1.255	GT41
EOGT	EGF domain-specific *O*-linked β-*N*-acetylglucosaminyltransferase	Extracellular *O*-GlcNAcylation	ER	2.4.1.255	GT61
DPAGT 1 Alg7	UDP-GlcNAc:dolichol-P GlcNAc-1-P transferase	*N*-glycosylation (first step)	ER	2.7.8.15	None
Alg13/Alg14	GlcNAc diphosphodolichol *N*-acetylglucosaminyltransferase	*N*-glycosylation (second step)	ER	2.4.1.141 3.4.19.12	GT1
MGAT1GnT-I GGNT1	Mannosyl (α-1,3-)-glycoprotein β-1,2-*N*-acetylglucosaminyltransferase	*N*-glycosylation (synthesis of hybrid and complex *N*-glycans)	Medial Golgi apparatus	2.4.1.101	GT13
MGAT2 GnT-II	Mannosyl (α-1,6-)-glycoprotein β-1,2-*N*-acetylglucosaminyltransferase	*N*-glycosylation (conversion of oligomannoses to complex *N*-glycans)	Golgi apparatus	2.4.1.143	GT16
MGAT3 GnT-III GGNT3	Mannosyl (β-1,4-)-glycoprotein β-1,4-*N*-acetylglucosaminyltransferase	*N*-glycosylation (bisecting GlcNAc)	Medial-trans Golgi apparatus	2.4.1.144	GT17
MGAT4 GnT-IV	Mannosyl (α-1,3-)-glycoprotein β-1,4-*N*-Acetylglucosaminyltransferase	*N*-glycosylation (synthesis of tri- and tetra-antennary *N*-glycans)	Golgi apparatus	2.4.1.145	GT54
MGAT5 GnT-V GGNT5	Mannosyl (α-1,6-)-glycoprotein β-1,6-*N*-acetylglucosaminyltransferase	*N*-glycosylation (initiation of β-1,6-branched structures)	Medial-trans Golgi apparatus	2.4.1.155	GT18
B3GNT8	UDP-GlcNAc: β-Gal β-1,3-*N*-acetylglucosaminyltransferase 8	*N*-glycosylation	Golgi apparatus	2.4.1.-	-
C2GnT GCNT1	core 2 β-1,6-*N*-acetylglucosaminyltransferase	Mucin-type *O*-glycosylation (synthesis of core 2)	Golgi apparatus	2.4.1.102	GT14
C3GnT B3GNT6	core 3 β-1,6-*N*-acetylglucosaminyltransferase	Mucin-type *O*-glycosylation (synthesis of core 3)	Golgi apparatus	2.4.1.149	GT31
C4GnT GCNT3	Core 2/Core 4 β-1,6-*N*-acetylglucosaminyltransferase	Mucin-type *O*-glycosylation (synthesis of cores 2 & 4)	Golgi apparatus	2.4.1.102	GT14
HAS1-3	Hyaluronic acid synthase 1–3	Hyaluronic acid synthesis	Plasma membrane (Cyt. face)	2.4.1.212	GT2
EXT1	Exostosin like glycosyltransferase 1	Heparin and heparan sulfate	ER	2.4.1.224	GT47
EXT2	Exostosin like glycosyltransferase 2	Heparan sulfate	ER and Golgi apparatus	2.4.1.224 2.4.1.225	GT47 GT64
EXT3	Exostosin like glycosyltransferase 3	Heparin and heparan sulfate	ER and Golgi apparatus	2.4.1.223	GT47
B3GNT5	UDP-GlcNAc: β-Gal β-1,3-*N*-acetylglucosaminyltransferase 5	Glycolipids (lacto and neolacto-series; crucial for Lewis X epitope)	Golgi apparatus	2.4.1.206	GT31
B3GNT8	UDP-GlcNAc: β-Gal β-1,3-*N*-acetylglucosaminyltransferase 8	*N*-glycosylation (elongation of branched structures)	Golgi apparatus	2.4.1.149	GT31
PIG-A/C/H/P/Q/Y	Phosphatidylinositol *N*-acetylglucosaminyltransferase subunits A, C, H, P, Q, and Y	GPI-anchors (synthesis of GlcNAc-phosphatidylinositol)	ER membrane (Cyt. face)	2.4.1.198	GT4

Cyt., cytosol; nucl., nucleus; mit., mitochondrion; ER, endoplasmic *reticulum*.

**Table 2:** Genes involved in glycosylation that are up- or down-regulated upon OGT depletion (from data published in Vella et al. 2013).

Symbol	Designation	Glycosylation Process	Subcellular Localization	EC Number	CAZy
**Upregulated**					
GLB1	β-galactosidase (beta 1)	Active on gangliosides, glycoproteins and GAG	Lysosome	3.2.1.23	GH35
FUT10	Fucosyltransferase 10 (α-1,3 fucosyltransferase)	Synthesis of Lewis X on *N*-glycans	Golgi apparatus	2.4.1.65	GT10
FUT8	Fucosyltransferase 8 (α-1,6 fucosyltransferase)	Active on complex *N*-type glycans	Golgi apparatus	2.4.1.68	GT23
MAN2A1	α-mannosidase, class 2A, member 1	Maturation of *N*-glycans	Golgi apparatus	3.2.1.114	GH38
MGAT5 GnT-V GGNT5	Mannosyl (α-1,6-)-glycoprotein β-1,6-*N*-acetylglucosaminyltransferase	*N*-glycosylation (initiation of β-1,6-branched structures)	Medial-trans Golgi apparatus	2.4.1.155	GT18
B4GALT6	UDP-Gal: βGlcNAc β-1,4 GalTase, polypeptide 6	Glycolipids (synthesis of lactosylceramide)	Medial-trans Golgi apparatus	2.4.1.274	GT7
B4GALT7	xylosylprotein β-1,4-galactosyltransferase, polypeptide 7	Proteoglycans	Golgi apparatus	2.4.1.133	GT7
B3GNT5	UDP-GlcNAc: β-Gal β-1,3-*N*-acetylglucosaminyltransferase 5	Glycolipids (lacto and neolacto-series; crucial for Lewis X epitope)	Golgi apparatus	2.4.1.206	GT31
UGGT1	UDP-Glc glycoprotein GlcTfase 1	*N*-glycosylation (glucosylation of unfolded proteins)	ER	2.4.1.-	GT24
GALNT1	UDP-N-GalNAc:polypeptide GalNAcTase 1 (GalNAc-T1)	*O*-glycosylation (mucin-type)	Golgi apparatus	2.4.1.41	GT27
GALNT10	UDP-N-GalNAc:polypeptide GalNAcTase 10 (GalNAc-T10)	*O*-glycosylation (mucin-type)	Golgi apparatus	2.4.1.41	GT27
GALNT12	UDP-N-GalNAc:polypeptide GalNAcTase 12 (GalNAc-T12)	*O*-glycosylation (mucin-type)	Golgi apparatus	2.4.1.41	GT27
**Downregulated**					
Alg14	Asn-linked glycosylation 14 homolog (*S. cerevisiae*)	*N*-glycosylation (second step)	ER	2.4.1.141	None
B4GALNT4	β-1,4-N-acetyl-galactosaminyltransferase 4	*N*-glycosylation	Golgi apparatus	2.4.1.244	GT7
OGT	*O*-linked β-N-acetylglucosaminyltransferase	*O*-GlcNAcylation	Cyt., nucl., mit.	2.4.1.255	GT41

Cyt., cytosol; nucl., nucleus; mit., mitochondrion; ER, endoplasmic *reticulum*.

**Table 3:** Main experimental evidences and speculations arguing for the regulation of complex glycosylations by *O*-GlcNAcylation.

Process	Reference
**Experimentally proved**	
***Nucleotide sugars levels***	
OGT and OGA interfere with UDP-Glc and UDP-HexNAc production	[20]
***Expression of enzymes of HBP***	
In *ogt-ko* animals, mRNAs encoding gfat2, gna-2, and the putative UDP-GlcNAc pyrophosphorylase C36A4.4 are up-regulated	[20]
UAP1 and Gnpda1 are upregulated in OGT NULL MEFs	[41]
***Transcriptional regulation***	
Transiently OGT-depleted mESCs exhibit either up- or down-regulation of genes involved in *N*- and *O*-glycosylations controlled by OGT	[48]
OGT regulates high-mannose *N*-linked glycans: OGT signaling in cholangiocarcinoma cells decreases MAN1A1 expression through a down-regulation of the MAPK-FOXO3 axis	[50]
***Protein synthesis through mTOR***	
ST3GAL6 expression correlates with mTOR activation in hepatoma carcinoma cells	[66]
ST8SIA4 expression is negatively correlated with mTOR activation in follicular thyroid cancer cells	[67]
**Speculative**	
***Nucleotide sugar levels***	
Competition for UDP-GlcNAc between OGT and other GTase (HAS, EOGT, reticular, and golgian GlcNAc transferases)	
***Transcriptional regulation***	
Transcriptional regulation of genes involved in glycosylation processes including nucleotide sugar transporters, GTases and GHases	
***Protein synthesis***	
Translation of glycosylation actors: Protection of eIF-2 by binding to *O*-GlcNAc forms of p67	[55,56]
OGT and OGA are partners of ribosomes; several ribosomal proteins are *O*-GlcNAcylated (e.g., RPS6)	[57]
Stabilization of nascent proteins by *O*-GlcNAcylation to prevent premature degradation	[59]
mTOR pathway is controlled by *O*-GlcNAcylation: Expression of glycosylation enzymes may be under the control of mTOR	[60,61,62]
***Vesicular traffic***	
Traffic of vesicular compounds through COPII	[85]
Through SEC23A, SEC24C, SEC31A, and TFG	[84,86]
Through the cytoskeleton	[87,88,89,90,91]
Through small G-proteins (Rab)	
